# The terrestrial green macroalga *Prasiola calophylla* (Trebouxiophyceae, Chlorophyta): ecophysiological performance under water-limiting conditions

**DOI:** 10.1007/s00709-016-1068-6

**Published:** 2017-01-09

**Authors:** Andreas Holzinger, Klaus Herburger, Kathrin Blaas, Louise A. Lewis, Ulf Karsten

**Affiliations:** 1 Functional Plant Biology, Institute of Botany, University of Innsbruck, Sternwartestrasse 15, 6020 Innsbruck, Austria; 2 Department of Ecology and Evolutionary Biology, University of Connecticut, Storrs, CT 06269-3043 USA; 3 Institute of Biological Sciences, Applied Ecology and Phycology, University of Rostock, Albert-Einstein-Straße 3, 18059 Rostock, Germany

**Keywords:** Chlorophyll fluorescence, Desiccation, Light, Photosynthesis, Temperature

## Abstract

The phylogenetic placement of *Prasiola calophylla*, from an anthropogenic habitat previously shown to contain a novel UV sunscreen compound, was confirmed by analysis of its *rbc*L gene. This alga has the capacity to tolerate strong water-limiting conditions. The photosynthetic performance and ultrastructural changes under desiccation and osmotic stress were investigated. Freshly harvested thalli showed an effective quantum yield of PSII [Y(II)] of 0.52 ± 0.06 that decreased to ∼60% of the initial value at 3000 mM sorbitol, and 4000 mM sorbitol led to a complete loss of Y(II). The Y(II) of thalli exposed to controlled desiccating conditions at 60% relative humidity (RH) ceased within 240 min, whereas zero values were reached after 120 min at 20% RH. All investigated samples completely recovered Y(II) within ∼100 min after rehydration. Relative electron transport rates (rETR) were temperature dependent, increasing from 5, 10, to 25 °C but strongly declining at 45 °C. Transmission electron microscopy of samples desiccated for 2.5 h showed an electron dense appearance of the entire cytoplasm when compared to control samples. Thylakoid membranes were still visible in desiccated cells, corroborating the ability to recover. Control and desiccated cells contained numerous storage lipids and starch grains, providing reserves. Overall, *P. calophylla* showed a high capacity to cope with water-limiting conditions on a physiological and structural basis. A lipophilic outer layer of the cell walls might contribute to reduce water evaporation in this poikilohydric organism.

## Introduction

Some green algae in the class Trebouxiophyceae (Chlorophyta), particularly members of the genus *Prasiola*, show a cosmopolitan distribution (Rindi 2007; Moniz et al. 2012; Heesch et al. 2016; Richter et al. 2016). The ecology of *Prasiola* is diverse, with some species growing in freshwater ecosystems or the supralittoral zone of marine coasts, and others occurring in terrestrial habitats (Friedmann 1969; Rodriguez et al. 2007; Rindi and Guiry 2004). Among terrestrial members, species such as *Prasiola crispa* ssp. *antarctica* even exhibit a symbiotic lifestyle with Ascomycota, forming the lichen *Mastodia tessellata* (Pérez-Ortega et al. 2010). In polar regions, *Prasiola* species prefer habitats rich in nitrogen and are found with faeces of birds such as penguins and growing in association with seagull colonies (Jackson and Seppelt 1997; Lud et al. 2001; Holzinger et al. 2006; Richter et al. 2016). Terrestrial *Prasiola* species are exposed to extremely harsh abiotic conditions, including strong water potential gradients between the soil surface and the surrounding atmosphere, resulting in regular desiccation that reduces the net production phases (Holzinger and Karsten 2013; Karsten and Holzinger 2014). In addition, terrestrial algae experience strong diurnal and seasonal fluctuations in insolation, including ultraviolet radiation (UVR). As a photoprotective mechanism under enhanced UVR, *Prasiola* species and related trebouxiophycean taxa synthesize and accumulate a natural sunscreen, a mycosporine-like amino acid (MAA) (e.g. Karsten et al. 2005). This compound, recently characterized from *Prasiola calophylla*, has a novel chemical structure composed of an oxo-carbonyl MAA (Hartmann et al. 2016). Given that prasiolin was described in *P. calophylla*, a detailed examination of the accompanying ecophysiology and cell structure/ultrastructure of this species during desiccation is needed.

The habitat occupied by *Prasiola* is an example of an ecological boundary between atmosphere and surface, in this particular case, a concrete wall covered by an extremely thin layer of soil. Boundaries tend to take on emergent properties that neither bordering region possess and will tend to modulate transfer of molecules and energy from one side to the other (Strayer et al. 2003). Many physiological activities and functions of terrestrial algae are related to trapping, transmission, transformation and/or loss of molecules across the atmosphere-soil interface that is occupied by these organisms. Water uptake and water loss represent key processes for the physiological activity of terrestrial algae.

Desiccation-tolerant terrestrial algae such as *P. crispa* ssp. *antarctica* often exhibit morphological adaptations, including thick cell walls that protect the protoplasts from mechanical damage and the capability to synthesize and accumulate sugar alcohols as ‘water-keeping’ substances (Jacob et al. 1991, 1992). Any damage due to desiccation limits the distribution of these species, and they usually grow in areas where at least occasional moisture is available. Damage caused by water-limiting conditions has recently been extensively reviewed for green algae (Karsten and Holzinger 2014; Holzinger and Pichrtová 2016). Fernandez-Marin et al. (2016) give a particular focus on the different photosynthetic mechanisms of desiccation-tolerant plant species.

The present study focused on the photophysiological and ultrastructural response of *P. calophylla* to naturally occurring water limitations in an extreme terrestrial habitat. Specifically, we investigated the (1) photosynthetic performance in response to cellular water loss (desiccation or osmotic stress) and (2) temperature dependence of photosynthesis, since the natural habitat has strong diurnally temperature changes due to varying sun exposures. Finally, we examined (3) the structural and ultrastructural features that might be responsible for the observed wide ecological tolerance. As this species is a promising model system for the physiological mechanisms of a terrestrial lifestyle, we also aimed to confirm its phylogenetic position by *rbc*L marker analyses. An unambiguous molecular taxonomic assignment is crucial, particularly in *Prasiola*, where morphological variation often occurs (Moniz et al. 2012; Heesch et al. 2016).

## Materials and methods

### Biological material

*Prasiola calophylla* (Carmichael ex Greville) Kützing (Trebouxiophyceae, Chlorophyta) was isolated repeatedly from the Botanical Garden of the University of Innsbruck (611 m above sea level, 47° 16′ 2″ N, 11° 23′ 34″ E) in July 2014, July 2015 and July 2016, as described by Hartmann et al. (2016) and either transferred to Storrs, Connecticut, USA, as life sample for DNA extraction, or immediately used for photophysiological, microscopic and transmission electron microscopic (TEM) examinations as described in the following sections. A voucher specimen of the sample used for DNA extraction was deposited at the George Safford Torrey Herbarium at the University of Connecticut (CONN accession no. 273714, barcode CONN00209659).

### DNA sequencing and phylogenetic analysis

Fresh *P. calophylla* material was extracted using the PowerPlant DNA Isolation Kit (MO BIO Laboratories, Inc., Carlsbad, CA, USA) according to the manufacturer’s protocol. Two primer sets were used for amplification and sequencing of the *rbc*L region, the PF2/PR2 primer pair (Rindi et al. 2004) and the SHF5/SHR8 pair (Heesch et al. 2012). The PCR mix consisted of 2 μL of extracted DNA, 10 μL of GoTaq® Green Master Mix, 0.5 μL of each primer (10 μM) and sterile deionized water for a final volume of 20 μL in the PCR tubes. The amplification cycle consisted of an initial denaturation step at 95 °C for 4 min, followed by 30 cycles of denaturation at 95 °C for 30 s, annealing at 47 or 50 °C for 30 s and extension at 72 °C for 1 min, with a final extension at 72 °C for 7 min. The sequencing reactions were performed using ABI PRISM BigDye Terminator Cycle Sequencing Kits and run on an ABI PRISM 3730XL Analyzer (Applied Biosystems). The sequences were verified in Geneious 9.0.5 (Biomatters, Auckland, New Zealand) and then used to prepare a consensus, which was subsequently aligned manually with the published *rbc*L sequences of related taxa. The appropriate substitution model was chosen using Modeltest in PAUP* 4.0a149 (Swofford 2002) using the Akaike information criterion (AICc). The best model selected under AIC was the general time reversible (GTR) + I + gamma. The model parameter values were set during the search based on a pilot analysis: RA-C = 1.0000, RA-G = 4.43327, RA-*T* = 1.0000, RC-G = 1.4440773, RC-*T* = 8.23139, RG-*T* = 1.0000, pinvar = 0.711319 and gamma shape = 0.937307 (four rate categories). A heuristic search with 100 random additions was performed to produce a ML tree in PAUP* using tree bisection-reconnection (TBR) branch swapping. Bootstraps analysis was performed using the same model parameters, with 100 replicates. Parsimony bootstrap analysis (1000 replicates) was performed, also in PAUP*, with TBR branch swapping. MrBayes 3.2.1 was used for the Bayesian analysis using the GTR + I + gamma model (Huelsenbeck and Ronquist 2001; Ronquist and Huelsenbeck 2003). Two independent Bayesian analyses were run for 5.1 × 106 generations with one cold plus three heated chains, with a subsample frequency of 1000. Convergence was determined, and trees from the initial 105 generations were discarded as burn in before producing the majority-rule consensus tree.

### Relative electron transport rates and temperature dependence

Relative electron transport rates (rETR) in response to increasing photosynthetically active radiation (PAR) up to ∼1600 μmol photons m^−2^ s^−1^ (PI curve) were monitored with a PAM-2500 chlorophyll fluorometer (Heinz Walz GmbH, Effeltrich, Germany) as previously described (Karsten et al. 2014; Herburger et al. 2015). Fresh algal material was harvested on a sunny day (July 2015) and PI curves (*n* = 4) were recorded at four different temperatures (5, 10, 20, 25 and 45 °C) in a KS-2500 suspension cuvette (Heinz Walz GmbH).

rETR curves were mathematically fitted with the photosynthesis model of Walsby (1997) to derive the alpha value (*α*; slope of the curve under light-limiting conditions), initial light saturation point (*I*_k_; μmol photons m^−2^ s^−1^) and maximum rETR (rETR_max_).

### Desiccation experiment and osmotic stress treatments

Desiccation experiments were performed in a desiccation chamber as previously described by Karsten et al. (2014), with modifications. *Prasiola calophylla* was either collected on a sunny dry summer day (July 2015) and exposed to 20 or 60% relative air humidity (RH) inside the chamber or collected on a rainy day during the same season and exposed to 20% RH. To generate water-limiting conditions via an extremely negative water potential, the samples were incubated in increasing concentrations of sorbitol (500, 1000, 2000, 3000 or 4000 mM) for 2 h according to the well-established approach of Kaplan et al. (2013) and references therein.

### Confocal and transmission electron microscopy

Confocal laser scanning microscopy (CLSM) was performed by using a Zeiss Pascal system (Carl Zeiss AG, Jena, Germany) on a Zeiss Axiovert 200 M. Chloroplast autofluorescence was visualized by exciting either fresh or desiccated thalli for 2.5 h (20% RH) at 488 nm and collecting emission above 560 nm (long pass filter, false color red). Corresponding bright field images were merged with autofluorescence images. Transmission electron microscopy was essentially performed as previously described (Holzinger et al. 2006). Freshly harvested samples were collected on a rainy day, mainly to illustrate the naturally fully hydrated condition, and either fixed directly or experimentally desiccated over a silica gel for 2.5 h prior to fixation. Briefly, samples were fixed in 2.5% glutaraldehyde in 50 mM cacodylate buffer for 1.5 h, rinsed and postfixed overnight in 1% OsO_4_ in the same buffer at 4 °C. Samples were then dehydrated in increasing ethanol concentrations, embedded in modified Spurr’s low viscosity resin sectioned and poststained with 2% uranyl acetate and Reynold’s lead citrate. Samples were viewed at a Zeiss Libra transmission electron microscope at 80 kV. Images were generated with a ProScan 2 k SSCCD camera and further processed with the Adobe Photoshop Elements 11 software (Adobe Systems, San José, CA, USA).

### Nile red staining

Freshly harvested *P. calophylla* thalli were washed in phosphate-buffered saline (PBS, 100 mM, pH = 7) and transferred to fresh PBS containing 0.1% (*v*/*v*) nile red (Sigma-Aldrich, Steinheim, Germany), which was previously dissolved in acetone (0.25% (*w*/*v*)). Staining was performed at an RT for 30 min. Thalli were washed 2× with PBS and investigated with a Zeiss Axiovert 200M equipped with an OSRAM HBO 50 Q/AC L1 CZ Mercury short ARC Photo optic lamp and Zeiss filter set 15 (excitation: BP 546/12 nm; emission: LP 590 nm).

### Statistical evaluation of the physiological data

Photosynthetic parameters derived from rETR curves at different temperatures (*α*, *I*_k_, rETR_max_; *n* = 4; *p* < 0.05), Y(II) values (*n* = 4; *p* < 0.01) under different sorbitol concentrations and Y(II) values (*n* = 5; *p* < 0.05) in response to desiccation/rehydration treatment were compared by one-way ANOVA followed by Tukey’s *post hoc* test. Data were analysed by Origin 8.5 (OriginLab Corporation, Northampton, MA, USA).

## Results

### Phylogenetic characterization

**Fig. 1:**
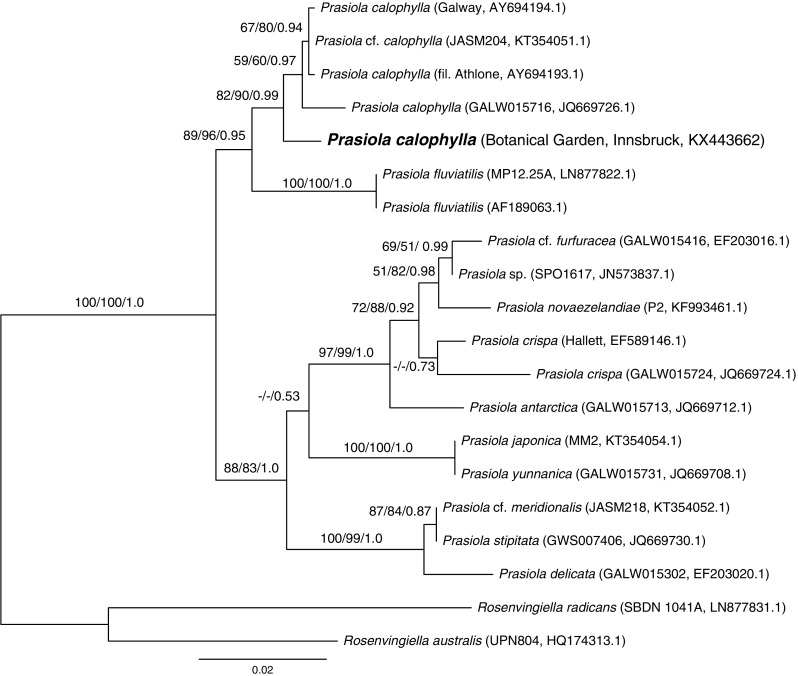
Inferred maximum likelihood tree (−lnL = 3430.617) of the *rbc*L sequence of the *Prasiola calophylla* investigated in this study and sequences from related taxa. Taxon labels include strain designation (where known and GenBank accession number). Node support values include ML bootstrap values/MP bootstrap values/Bayesian Posterior Probabilities. *Scale bar* indicates expected number of substitutions per site

The resulting *rbc*L sequence is 1180 nucleotides in length and was deposited in GenBank under accession no. KX443662. Phylogenetic analysis of the *rbc*L data from the Botanical Garden specimen supports designation of this alga as a member of *P. calophylla* along with specimens from Ireland and Japan (Fig. 1). The *rbc*L sequence of the focal specimen is quite similar to other accessions of *P*. *calophylla*, differing by just 9–15 nucleotide differences, all representing synonymous changes, and the next closest species to any included sequence of *P. calophylla* is *Prasiola fluviatilis*, with 26–31 nucleotide differences (not shown).

### Temperature-dependent photosynthetic performance

**Fig. 2:**
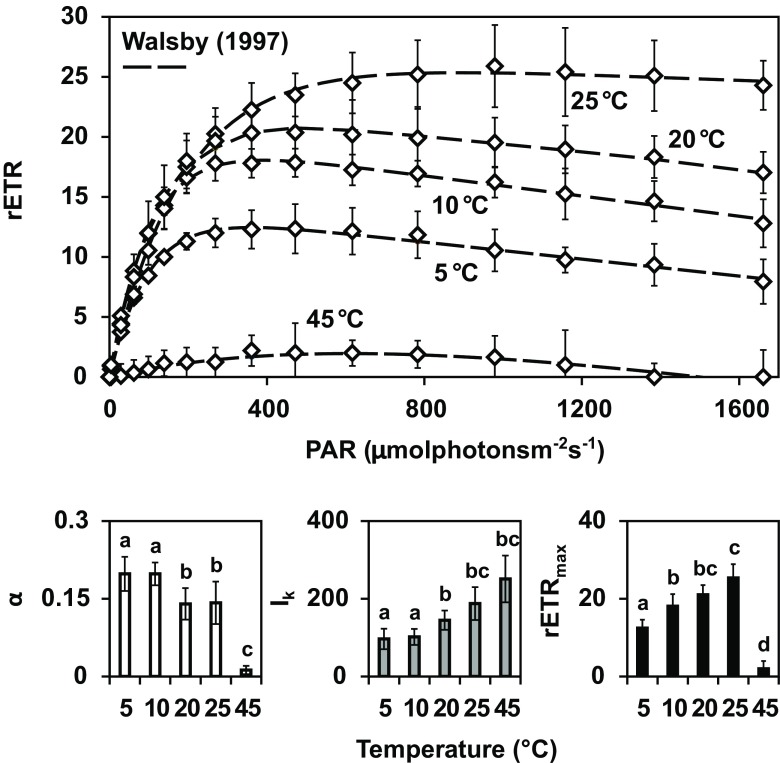
Relative electron transport (rETR) rates (*n* = 4 ± SD) in response to increasing PAR up to ∼1600 μmol photons m^−2^ s^−1^ at 5, 10, 25 and 45 °C. Light curve parameters are given in a temperature-dependent manner, *α* (initial slope of the curve under light-limiting conditions), *I*_k_ (initial light saturation point) and maximal rETR (rETR_max_). Values are means of *n* = 4, and statistically significant subgroups (*p* < 0.05) are marked with *lowercase letters*

Samples were isolated on a sunny day (26 °C, cloud cover: 31%, RH: 43%, rainless during the last 24 h). rETR curves recorded at 5 and 10 °C showed similar *α* (∼0.2) and *I*_k_ values (∼100 μmol photons m^−2^ s^−1^), indicating similar kinetics (Fig. 2). At 20 and 25 °C, *α* decreased significantly (*p* < 0.05), while the *I*_k_ value increased significantly (*p* < 0.05) to ∼200 μmol photons m^−2^ s^−1^ at 25 °C (Fig. 2). The rETR_max_ values increased linearly from 5 to 25 °C (optimum) with values above 20 °C, while rETR_max_ was strongly inhibited at 45 °C (Fig. 2). As suggested by slightly declining rETR above 400 μmol photons m^−2^ s^−1^, moderate photoinhibition was present at 5 and 20 °C, but absent at 25 °C (Fig. 2).

### Desiccation and rehydration kinetics

**Fig. 3:**
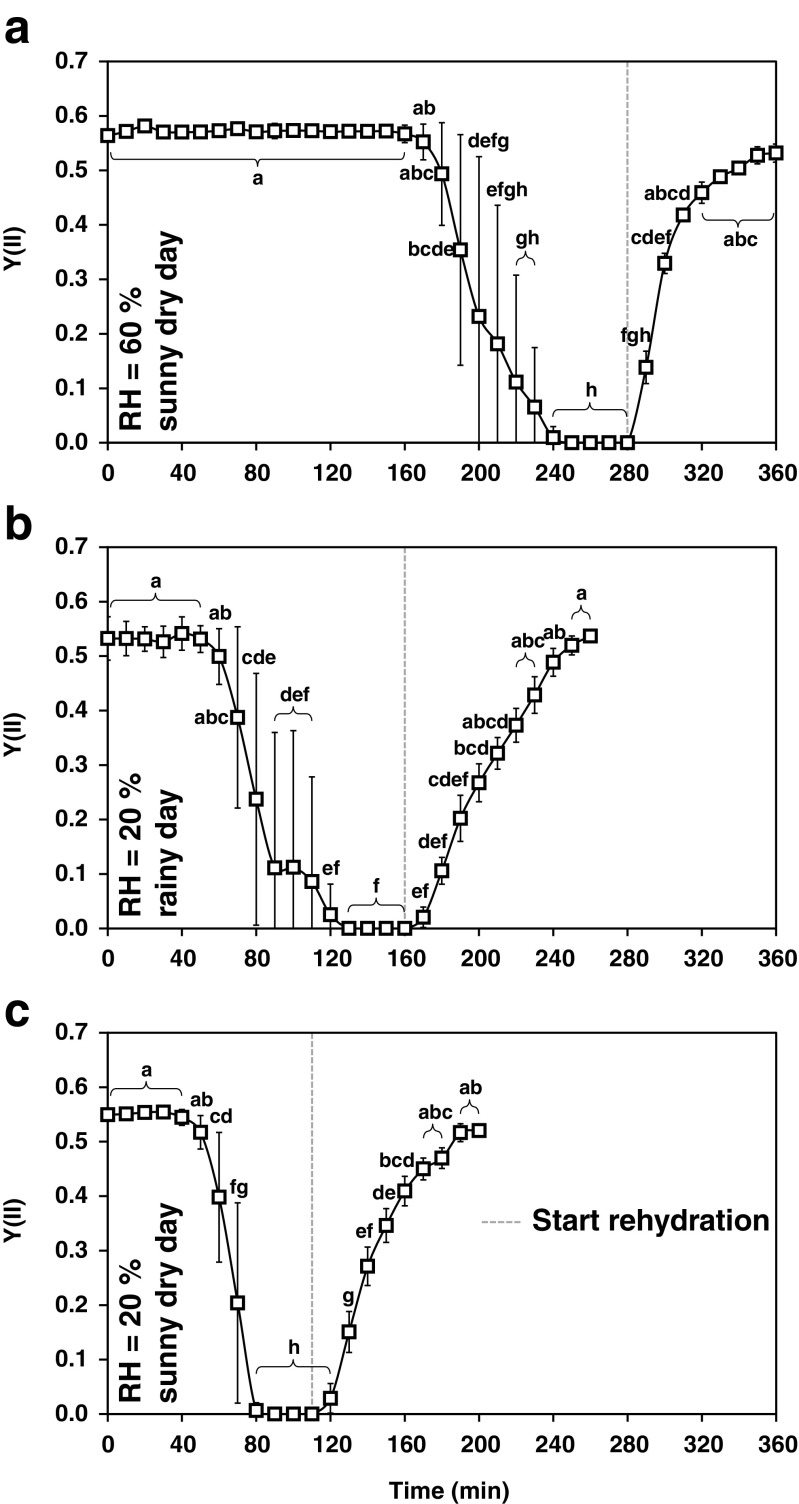
Effective quantum yield of PSII (*n* = 5 ± SD) during desiccation and rehydration. **a** Samples harvested on a sunny and dry day and exposed to a relative humidity (RH) of 60%. **b** Samples collected on a rainy day and desiccated at 20% RH. **c** Samples collected on a sunny day and exposed to ∼20% RH. The *dashed grey lines* mark the start of rehydration. *Lowercase letters* indicate statistically different subgroups as determined by one-way ANOVA followed by Tukey’s *post hoc* test (*p* < 0.05)

*P. calophylla* harvested on a sunny day (28 °C, cloud cover: <20%, RH: 53%, rainless during the last 24 h) showed an initial effective quantum yield of PSII [Y(II)] of 0.57. After ∼180 min of desiccation at 60% RH, the Y(II) decreased to zero within 1 h (Fig. 3a). After another 40 min, thalli were rewetted, which caused a rapid increase of the Y(II) with full recovery of the effective quantum yield within ∼1 h. Thalli collected on a rainy day [20 °C, cloud cover: 84%, RH: 81%, rainfall during the last ∼36 h] and exposed to desiccation over a silica gel at ∼20% RH started to decrease their initial Y(II) of 0.53 after 1 h, whereas complete inhibition of effective quantum yield was reached after ∼2 h (Fig. 3b). Rehydration after 40 min increased the Y(II) linearly to the initial value within ∼2 h. When a similar experiment (desiccation at 20% RH) was performed with samples collected on a sunny day (31 °C, cloud cover 0%, RH: 16%, rainless during the last 24 h), the Y(II) value (0.55) decreased more rapidly to zero (within ∼40 min). However, full recovery of the photosynthetic performance still occurred within ∼2 h (Fig. 3c).

### Water potential effects

**Fig. 4:**
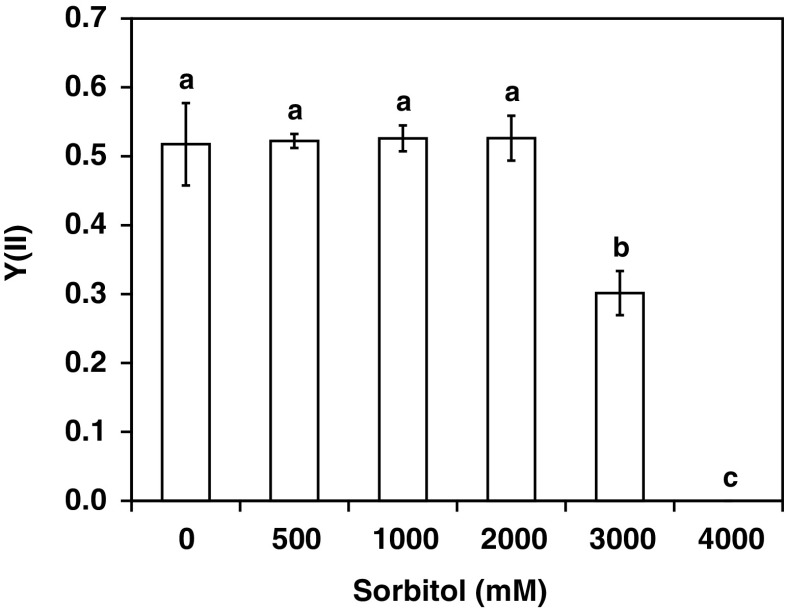
Effective quantum yield of PSII (*n* = 4 ± SD) of *Prasiola calophylla* exposed to increasing concentrations of sorbitol. Statistically significant differences were determined by one-way ANOVA followed by Tukey’s post hoc test; significantly different subgroups (*p* < 0.01) were marked with *lowercase letters*

Thalli exposed to osmotically active sorbitol solutions up to 2000 mM for 2 h showed no significantly different Y(II) values (Fig. 4). At 3000 mM sorbitol, the initial Y(II) dropped significantly (*p* < 0.01), while at 4000 mM sorbitol, no Y(II) signal was measurable (Fig. 4).

### Light and confocal microscopic observations

**Fig. 5:**
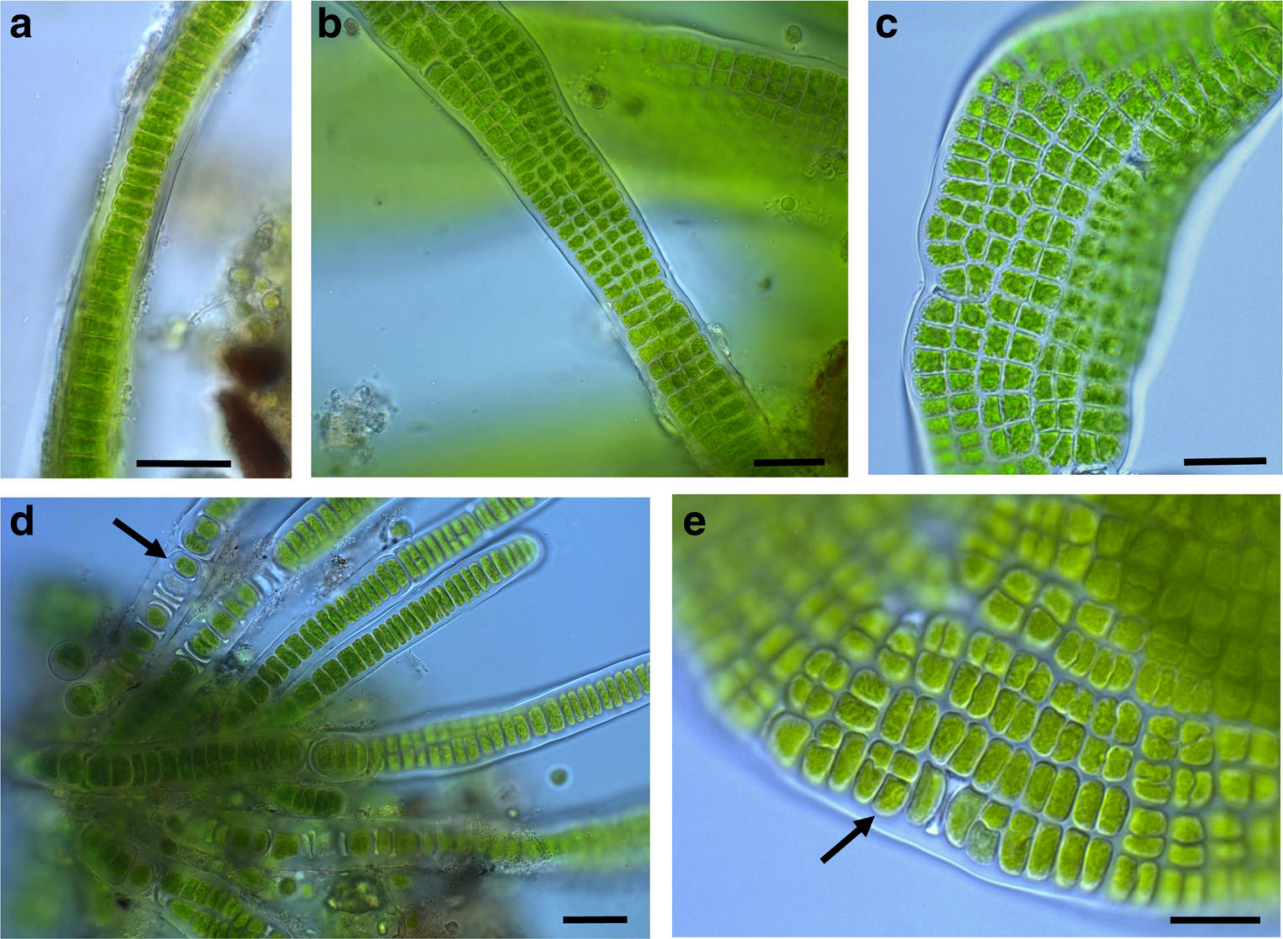
Light micrographs of *Prasiola calophylla*. **a** Uniserate filament. **b** Filament consisting of four cell rows. **c** Thallus exposed for 2 h in 1000 mM sorbitol; no visible change to untreated filament. **d** Uniserate thalli of the base treated for 2 h in 3000 mM sorbitol; only slight rounding of the protoplasts visible. **e** Thallus exposed for 2 h in 4000 mM sorbitol; plasmolysis is visible by slightly rounded protoplasts. *Bars* **a**–**d** 20 μm, **e** 10 μm

*Prasiola calophylla* showed a mixture of uniseriate (Fig. 5a) and multiseriate (Fig. 5b) filaments of varying diameters of up to 250 μm. The cells were usually arranged in rows. Cells exposed to hypertonic sorbitol solutions of 1000 mM did not show any visible change of the protoplasts (Fig. 5c). Basal uniseriate filaments exposed to 3000 mM sorbitol were only slightly affected, with several filaments appearing similar to the control cells, with just a few filaments plasmolysed (Fig. 5d). Only incubation at 4000 mM resulted in rounded and detached protoplasts in all cells (Fig. 5e).

**Fig. 6:**
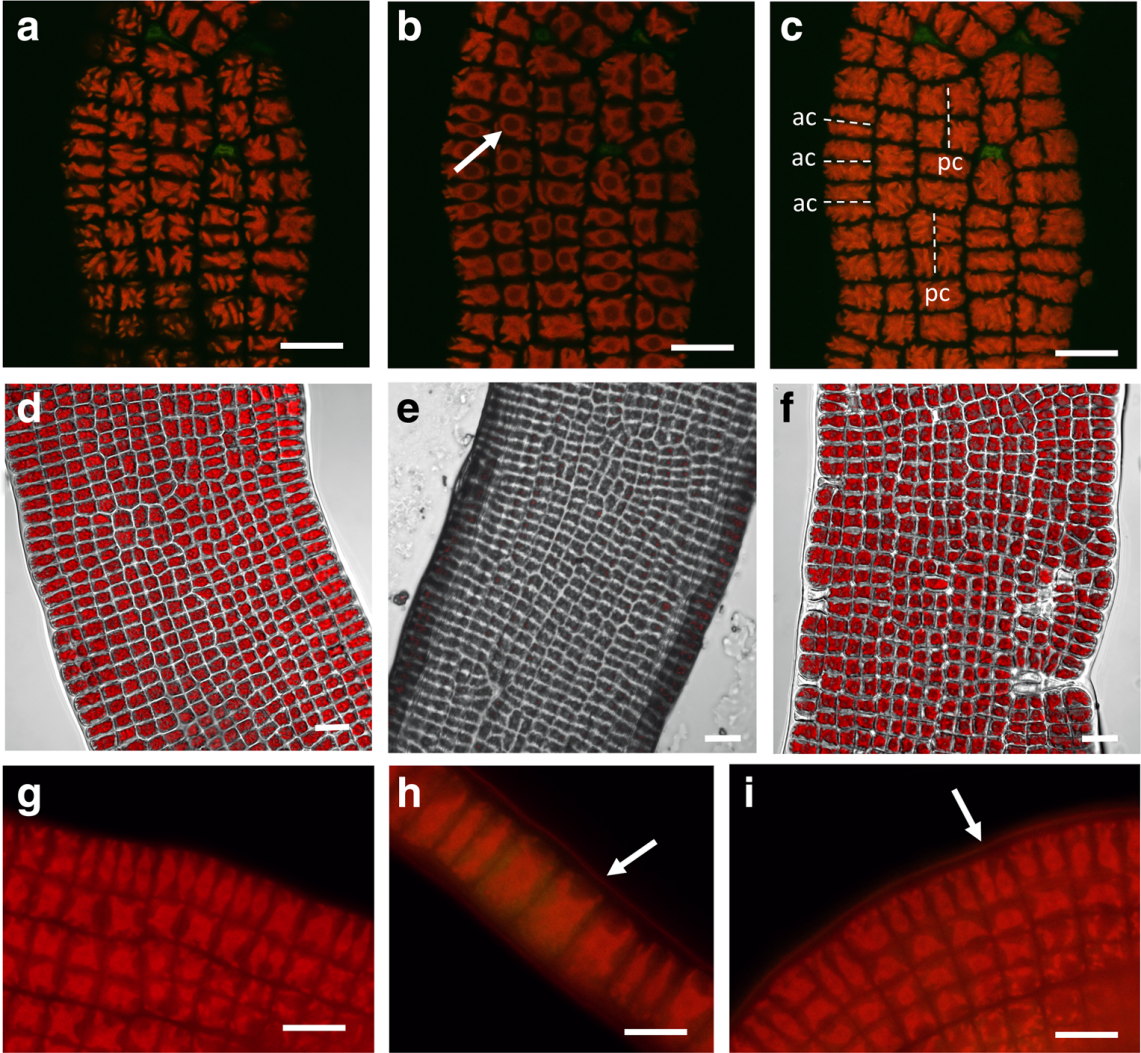
Confocal laser scanning microscopy (**a**–**f**) and epifluorescence microscopy (**g**–**i**) of *Prasiola calophylla*. **a** Cortical optical section of chloroplast lobes. **b** Median optical section showing the round pyrenoids free of chlorophyll autofluorescence. **c** z-stack projection allowing to depict the division planes of the cells. *ac* anticlinal cell division, *pc* periclinal cell division. **d** Freshly harvested thallus segment illustrating the cell walls and the chloroplast autofluorescence. **e** Segment of the same thallus desiccated for 2.5 h at 20% RH; chlorophyll autofluorescence (*false color red*) declined strongly. **f** Segment of the thallus rehydrated for 1.5 h. **g** Control thallus segment where nile red staining was omitted. **h** Uniserate filament stained with 0.1% nile red. **i** Thallus segment stained with 0.1% nile red, showing reddish fluorescence labelling on the surface of the thallus (*arrows*). *Bars* 10 μm

When observed with CLSM, the chloroplast lobes were clearly visible by chlorophyll autofluorescence, with surface sections showing multiple lobes (Fig. 6a). Median optical sections allowed visualization of the central pyrenoid, which appeared as round structures free of chlorophyll autofluorescence (Fig. 6b). The z-stack projection clearly shows the division planes of individual cells in a thallus segment. Anticlinal divisions, which elongate the filament, are perpendicular to the thallus surface, whereas periclinal cell divisions lead to thallus broadening (Fig. 6c). Figure 6 (d–f) shows thallus segments, where a bright field image (grey) is merged with an image showing the chlorophyll autofluorescence (red). Desiccation for 2.5 h over a silica gel resulted in an almost complete loss of the visible chlorophyll autofluorescence (Fig. 6e); the signal was, however, recovered fully, when thalli were rewetted for 2 h (Fig. 6f). Epifluorescence visualization of control thalli also clearly showed the chloroplast autofluorescence (Fig. 6g), and nile red staining of uniseriate filaments had reddish fluorescence on the thallus surface (Fig. 6h), also seen in mature, broader thalli (Fig. 6i).

### Transmission electron microscopy of control and desiccated cells

**Fig. 7:**
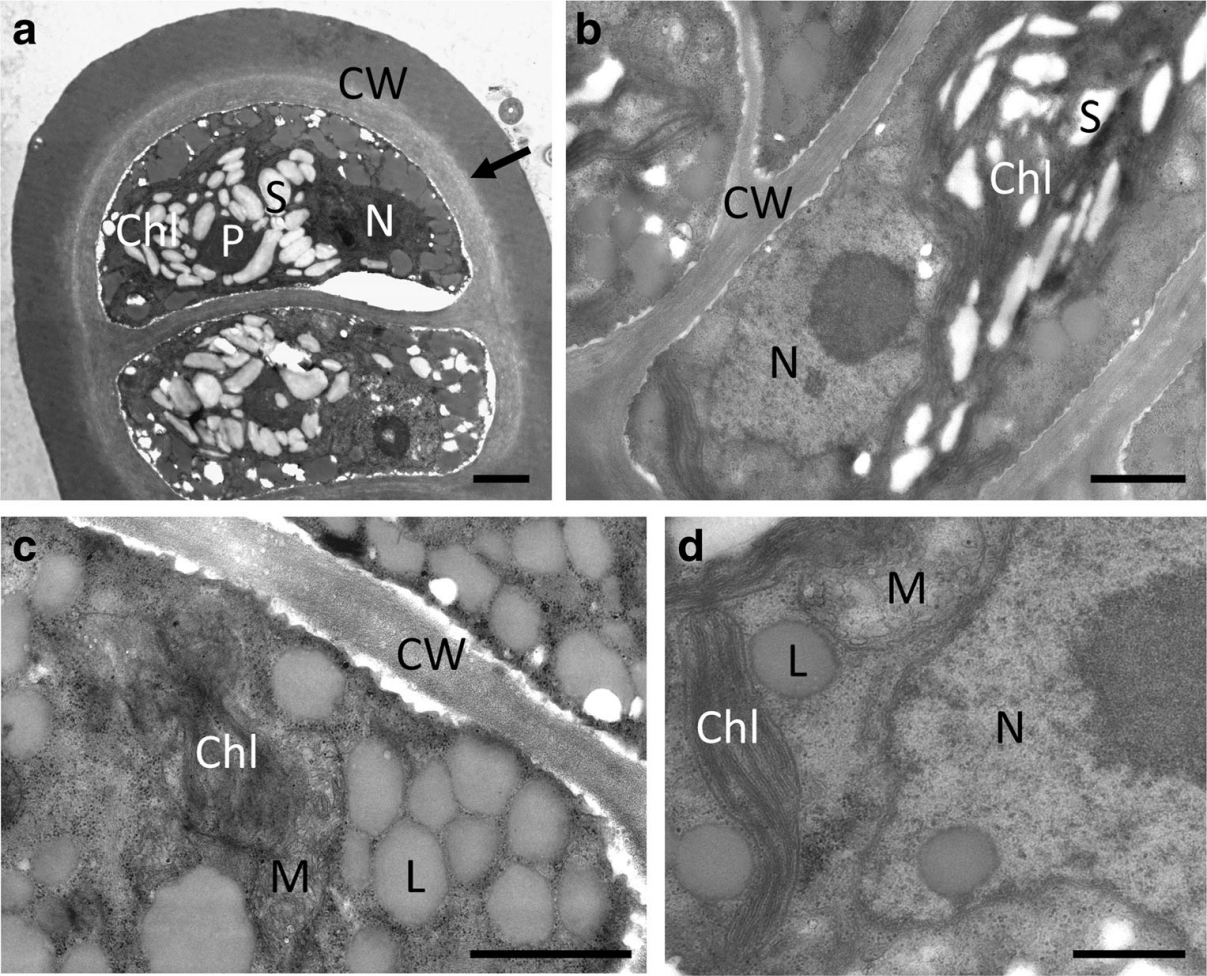
Transmission electron micrographs of *Prasiola calophylla* control cells. **a** Terminal cells of uniseriate filament, dense cytoplasm and numerous starch grains around the pyrenoid: notice the bi-layered cell wall (*arrow*). **b** Top view on thallus segment, starch grains in chloroplast. **c** Numerous lipid bodies in the cell periphery. **d** Detail view of an area close to the nucleus, chloroplast lobe with thylakoid membranes, lipid bodies and mitochondrion. *Chl* chloroplast, *CW* cell wall, *L* lipid body, *M* mitochondrion, *N* nucleus, *P* pyrenoid, *S* starch grain. *Bars* **a** 2 μm. **b**, **c** 1 μm. **d** 0.5 μm

**Fig. 8:**
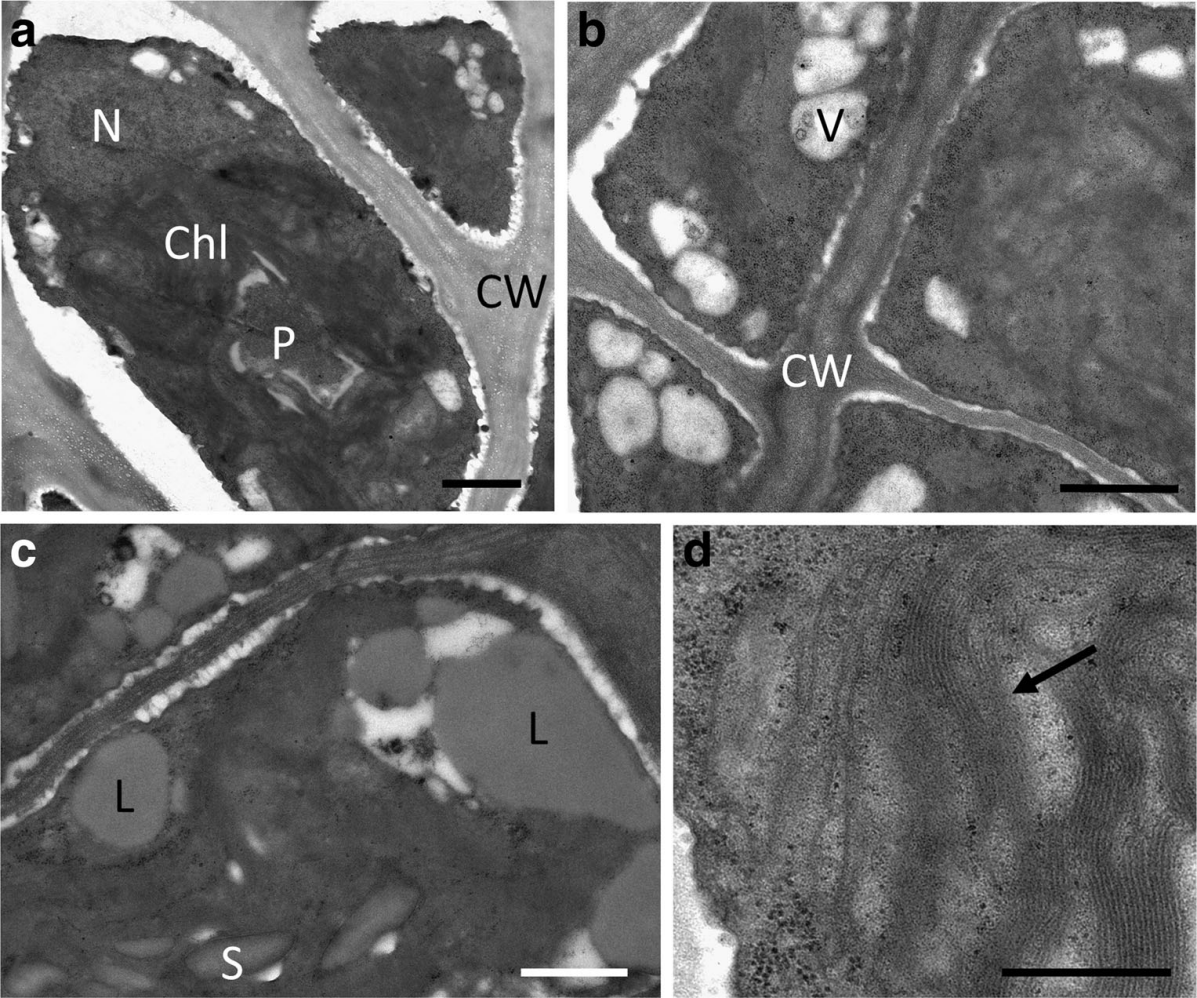
Transmission electron micrographs of filed-collected *Prasiola calophylla * samples desiccated at ∼20% RH for 2.5 h. **a** Dense cytoplasm slightly detached from the cell wall, pyrenoid clearly visible. **b** Cells with homogenous cell content, small vacuoles in the cell cortex. **c** Detail of a cell containing large lipid bodies and starch grains. **d** Detail of the chloroplast with still intact thylakoid membranes (*arrow*). *Chl* chloroplast, *CW* cell wall, *L* lipid body, *N* nucleus, *P* pyrenoid, *S* starch grain. *Bars* **a**–**c** 1 μm, **d** 0.5 μm

Transmission electron microscopy of *P. calophylla* cells revealed a dense cytoplasm that was virtually vacuole free (Fig. 7a–d). The terminal cells showed a thick bi-layered cell wall (Fig. 7a). The cells contained a marked pyrenoid surrounded by numerous starch grains (Fig. 7a, b). Inside the thallus, the cell walls appeared much thinner, most likely a consequence of recent cell divisions. The cells showed numerous lipid bodies in the cell periphery (Fig. 7c) and in the centre (Fig. 7d). Mitochondria and chloroplast lobes were clearly detected (Fig. 7d). Cells desiccated for 2.5 h showed more electron density of the protoplasts, and occasionally, the cytoplasm was markedly detached from the cell walls. The chloroplast and nucleus were still clearly visible (Fig. 8a), and the structure within the chloroplasts was very dense (Fig. 8b, c), but occasionally still showed intact thylakoid membranes (Fig. 8d). Numerous small vacuoles were visible (Fig. 8b), and large lipid bodies were found in the cell periphery (Fig. 8c).

## Discussion

In the present study, the photophysiological performance of *P. calophylla* was investigated in response to water-limiting conditions as well as ultrastructural changes upon severe desiccation. Moreover, a phylogenetic characterization was performed based on the *rbc*L gene analysis, confirming the previous morphological taxonomic characterization (Hartmann et al. 2016). This species is especially interesting ecologically, as an abundant occurrence of a new 324-nm absorbing UV sunscreen—termed ‘prasiolin’—was isolated and chemically characterized in natural samples from the same locality (Hartmann et al. 2016). This photoprotectant likely contributes to the ecological success of *P. calophylla* in aeroterrestrial habitats.

Ephemeral *Prasiola* species have the capability to grow outside the aquatic milieu on bark, soil and rock and in the supralittoral zone of marine rocky shores (Rindi et al. 2007). This broad ecological amplitude makes this genus particularly interesting to study physiological adaptations to terrestrial habitats, which base either one a freshwater or a marine lifestyle. As far as known, the terrestrial members of this genus always seem to prefer nitrophilic environments shaped by bird faeces, as reported for Antarctic and Arctic taxa (Holzinger et al. 2006; Kosugi et al. 2010; Richter et al. 2016). This is confirmed for *P. calophylla* of the present study, which benefits from dog excrements (Hartmann et al. 2016). Nitrogen is essential not only for growth but also for the biosynthesis of the N-containing UV sunscreen prasiolin. A similar nitrogen dependence for the production of UV-protecting substances has been shown in the red alga *Porphyra* (Korbee et al. 2005).

### Phylogenetic position and morphological characteristics

Kützing (1848) originally described *P. calophylla* (Carmichael ex Greville) Kützing from Argyll, Scotland, as dark green, curly and almost linear or ribbon shaped, with filaments gradually narrowing toward the base (Greville 1826). The species is found in cold temperate regions of the Northern Hemisphere (Guiry and Guiry 2016) and was recently reported from a wharf in Japan (Sutherland et al. 2016). Several Antarctic records of this species exist; however, Moniz et al. (2012) indicated that the *rbc*L sequences of these specimens diverged by 38–42 bp from European specimens of *P. calophylla*, far greater than the variation even between the accessions from northern Europe and Japan. Moreover, a significantly different habitat (stream- and water-flushed ground distant from fertilization by birds) was described for the Antarctic collections by Broady (1989). *Prasiola calophylla* was so far only confirmed from northeastern Atlantic and central European regions (Heesch et al. 2016). The position of *P. calophylla* as sister taxon to *P. fluviatilis* was also confirmed by Heesch et al. (2016). Thus, the earlier reports of *P. calophylla* from Antarctica have to be questioned.

The chloroplast morphology was determined by CLSM, as due to the dense cytoplasmic arrangement, it was difficult to observe the structure of the chloroplasts by light microscopy alone; particularly at the CLSM images, the division planes at the sides of the thalli were clearly visible, while anticlinal cell divisions took place at the cell periphery, leading to an elongation of the blades; periclinal cell divisions were observed more toward the centre of the blades. These periclinal cell divisions lead to broadening of the thalli; this division mode is basically the same for all *Prasiola* sp.; however, the regularity of the occurring of periclinal cell divisions leads to different degrees of curling (Richter et al. 2016).

### Water-limiting conditions

To investigate the effect of experimentally applied water-limiting conditions, *P. calophylla* was either exposed to osmotically active sorbitol (Kaplan et al. 2012, 2013) or to 20% respectively 60% RH, resulting in desiccation. The small thalli collected in the field were densely packed with cytoplasm, exhibited a very low degree of vacuolization and required extremely high concentrations of sorbitol to induce incipient plasmolysis. In fact, plasmolysis in *P. calophylla* occurred only when exposed to 3000–4000 mM sorbitol. These concentrations translate into very low water potentials of −13 to −23.3 MPa (Kosugi et al. 2014). Y(II) was decreased to ∼60% of the initial value at 3000 mM, and at 4000 mM, Y(II) was completely suppressed. Similarly, in an Antarctic *P. crispa*, the effective quantum yield of PSII reached zero at 3000 mM sorbitol (Kosugi et al. 2010). In *Trentepohlia aurea* (Ulvophyceae) and some chlorolichens (e.g. *Stereocaulon sorediiferum*), however, electron flow through PSII remains active even at 4000 mM sorbitol (Kosugi et al. 2010, 2014).

In contrast, the aeroterrestrial streptophyte green alga *Klebsormidium* sp. was plasmolysed at 600–800 mM sorbitol (Kaplan et al. 2012). When compared with aquatic algae, even this value can be regarded as high. Moreover, in the late diverged streptophyte green alga *Zygnema* sp., which prefers hydroterrestrial habitats, incipient plasmolysis was found between 400 and 600 mM sorbitol. These data suggest that *P. calophylla* exhibits a constitutively very negative intracellular osmotic potential, reducing cellular water loss due to desiccation. The major inorganic and organic solutes that might contribute to the osmotic potential have been described as potassium, phosphate, amino acids and low molecular weight carbohydrates in *P. crispa* ssp. *antarctica* (Jacob et al. 1991). Another interesting phenomenon has been observed under high salinity treatments of *P. crispa* ssp. *antarctica*, which lead to the occurrence of vacuoles that regulate the volume change of the cells (Jacob et al. 1991); this phenomenon appears contradictory at the first glance, as one would expect water loss from the vacuoles. Yet, the mechanical benefit of this capacity was described in *Porpyhra umbilicalis*, which uses these vacuoles to mechanically avoid separation of the protoplasm from the cell wall (Wiencke and Läuchli 1981; Wiencke et al. 1984).

### Photosynthetic performance

To complement osmotic experiments, we analysed the physiological parameters by continuous measurements of the effective quantum yield in field samples which were either collected on rainy or sunny days and exposed to ∼20% or ∼60% RH, respectively. The desiccation kinetics revealed two factors influencing the Y(II) of *P. calophylla* under water scarcity: (1) moist conditions (i.e. rainfall, high RH) prior to desiccation stress increased the maintenance of photosynthetic activity during desiccation, whereas a previous dry period caused an earlier inhibition. (2) Increasing the RH to ∼60% maintained the Y(II) for a longer interval despite a previous dry weather period. This suggests that short-term acclimation to dry conditions (i.e. hardening) plays only a minor role in *P. calophylla* to cope with low water availability, since dry weather periods do not increase the desiccation tolerance. Therefore, some constitutive tolerance mechanisms, such as an extremely negative osmotic potential inside the cells, might be particularly important to survive severe desiccation stress and to recover quickly after rehydration. This includes loss of PSII reaction centre activity combined with a strong reduction of both the photo-oxidation state and rereduction of PSI under dry conditions as suggested for *P. crispa* from an Antarctic habitat (Kosugi et al. 2010). Unlike light-induced NPQ, this desiccation-induced quenching is not restricted to light, as it also occurs in the dark (Nabe et al. 2007). It coincides with a strong reduction of fluorescence emitted between ∼680 and ∼780 nm (Kosugi et al. 2010), which was also indicated in the present study by visualizing the reduction of the chlorophyll autofluorescence, illustrated by confocal laser scanning microscopy where we choose 20% RH, to gain maximal desiccation. However, the chlorophyll autofluorescence increased again upon rehydration, indicating the recovery of this treatment. Analogous effects, i.e. the inactivation of PSII while PSI still maintains activity, were observed in *Ulva* sp., where PSI remained even active after exposure to 4000 mM sorbitol (Gao et al. 2011).

However, at the present state, we can only speculate about the extremely well performance under water-limiting conditions in *P. calophylla*; possible candidates for protection are, e.g. flavodiiron proteins, which are involved in cyanobacterial energy dissipation in photosynthesis; Flv1 and Flv3 proteins, functioning in the ‘Mehler-like’ reaction that protects PSI, have been found not only in cyanobacteria but also in green algae, mosses and lycophytes (e.g. Allahverdiyeva et al. 2015). Moreover, genes coding for flavodoxin, which is induced in cyanobacteria and green algae under osmotic-, heat- and high light stress, have been found in many Trebouxiophyceae (Karlusich et al. 2014). However, flavodoxins were lost during evolution and are lacking in streptophytic green algae and plants and tend to be lost in iron-rich habitats. In contrast, ferredoxins have been maintained throughout plant evolution. They are electron shuttlers harbouring iron-sulfur clusters and are particularly sensitive to desiccation, leading to downregulation of photosynthesis in streptopyhte green algae (e.g. Karlusich et al. 2015). However, up to now, only the draft plastid and mitochondrial genome sequences from *P. crispa* have been published (Carvalho et al. 2015); therefore, no direct evidence on the occurrence of these protection mechanisms in *Prasiola* is available.

### Ultrastructural changes

The ultrastructural changes upon severe desiccation for 2.5 h corroborate our observations obtained by desiccation experiments. Untreated control cells already had a very dense cytoplasm, typical for terrestrial *Prasiola* sp. (e.g. Holzinger et al. 2006; Richter et al. 2016). This was even the case in the fully hydrated natural condition, as the samples for TEM were harvested on a rainy day. Interestingly, even under severe desiccation conditions (20% RH for 2.5 h; we choose this treatment to illustrate the effect of the maximal water loss), some thylakoid membranes remained intact, suggesting molecular protection mechanisms as realized in other green algae (Gasulla et al. 2013). However, the preservation of the ultrastructure of desiccated samples by chemical fixation has its limitations (see Holzinger et al. 2011); for future studies, high pressure freeze fixation followed by freeze substation should be applied, but even with this technique, it is not possible to freeze desiccated samples, and an intermedium (e.g. sucrose solution) has to be used. Given the fact of the extremely negative osmotic potential, rehydration prior to the freezing process could hardly be avoided. A similar condensed cytoplasm was observed in other green algae (Holzinger et al. 2011) or mosses (Pressel and Duckett 2010). A particularly interesting observation is the occurrence of vacuoles in 2.5 h desiccated cells. As discussed previously for *P. crispa* under osmotic stress (Jacob et al. 1992), the same phenomenon also occurs under desiccation; in *P. crispa*, it was assumed that the vacuoles are deposition sites for ions which enter the cells. Severe desiccation goes along with changes in the ionic homeostasis that needs to be buffered to some extent, and these emerging vacuoles might be a way to compensate for these increases.

### Contribution of the cell walls

Furthermore, the cell walls of *P. calophylla* withstand the mechanical stress induced by desiccation, and the cytoplasm appears only slightly retracted in some parts. However, this was also the case in the non-desiccated group. In *P. calophylla*, we did not observe the desiccation-induced cell wall undulations observed in *Klebsormidium* sp. (Holzinger et al. 2011; Herburger and Holzinger 2015) or any inner pectic layers as in *Ulva compressa* (Holzinger et al. 2015). The thalli of *U. compressa* and *P. calophylla* show some similarities, such as a flat leaf-like appearance and highly ordered arrangement of cells. However, the morphological responses to desiccation stress followed by rehydration are different in the different genera, likely due to different cell wall architectures and chemistries. Individual *Ulva* cells are surrounded by a flexible pectic layer, followed by several cellulose- and xyloglucan-rich layers, which are embedded in an amorphous matrix consisting of pectins and ulvan (Lahaye and Robic 2007). This allows both, the protoplasts and the whole thallus to shrink and expand reversibly upon desiccation and rehydration, respectively, which minimizes structural und ultrastructural damage (Holzinger et al. 2015). In contrast, Trebouxiophyceae lack common pectic polymers found in *Ulva* (e.g. homogalacturonan; Domozych et al. 2012) and desiccation for up to 2.5 h at 20% RH did not deform thalli of *P. calophylla* significantly. As shown for *Prasiola japonica*, *Prasiola* cell walls consist of at least four chemically distinguishable layers (Takeda et al. 1967). Interestingly, the innermost layer surrounding the protoplast contains mainly cellulose, followed by a thick and stable mannan-rich layer, while acidic polysaccharides (e.g. uronic acid) are restricted to thin layers in anticlinal walls between individual cells (Takeda et al. 1967). Embedding the protoplasts and individual cells into a rigid matrix appears to reduce mechanical deformation upon water loss and restricts structural damage to a minimum as shown for rehydrated thalli. In combination with a high water holding capacity of the protoplasts due to low osmotic pressures, structural damage as a consequence of water scarcity can be minimized. Thalli of *U. compressa* did not recover their photosynthetic performance after 90 min of desiccation at ∼62% RH, corresponding to 27% relative water content (RWC) of the thalli. Although the RWC was not measured in *P. calophylla*, we can estimate that in the present study, we have more severely desiccated the *P. calophylla* thalli, since they were exposed to ∼20% RH for over 2.5 h. Despite maintaining the desiccated state for at least 40 min, all samples completely recovered.

*Prasiola crispa*, occurring in the littoral zone of the Antarctic sea, was able to survive a water loss of 86% after desiccation at 50% RH for 14 days (Jacob et al. 1992). In addition to very low osmotic pressure inside the protoplast, the high water holding capacity of *Prasiola* thalli might also be attributed to a cuticle-like layer on the thallus surface, as shown by nile red staining, a fluorescence dye commonly used to visualize neutral lipids being part of cuticles (Broehan et al. 2013). As shown for the brown alga *Fucus distichus*, this cuticle-like layer might consist of unsaturated fatty acids (Quadir et al. 1979). Interestingly, in *P. crispa*, a relation between excessive lipid bodies and fatty acids incorporated into the cell wall was assumed (Jacob 1992); since these cytoplasmic lipid bodies remain stable even after cultivation for several months in the dark, a functional role as storage compounds is unlikely. Instead, they might contain the building blocks for the cuticle-like layer. This hypothesis is supported by the observation that *Prasiola* species occurring in terrestrial or littoral habitats, where desiccation stress occurs frequently, usually contain abundant lipid bodies (this study; Jacob et al. 1992; Holzinger et al. 2006). Accumulating lipid bodies functioning as storage compounds in green algae is usually related to nitrogen deprivation (Pichrtová et al. 2014), which can be excluded for the organism investigated in the present study, since a high nitrogen input due to dog excrements is considered to contribute to the abundant growth (Hartmann et al. 2016). Nitrogen is also essential for biosynthesis of the N-containing UV sunscreen prasiolin (Hartmann et al. 2016).

### Temperature dependence

Besides water availability, the temperature regime in the habitat strongly influences the photosynthetic performance of terrestrial algae, with low temperatures reducing the catalytic activity of enzymes involved in the Calvin-Benson cycle. As a consequence, electron carriers of the photosynthetic apparatus might be overreduced, which leads to a reduction of the photosynthetic performance. In *P. calophylla*, the highest rETR in response to increasing photon fluence rates occurred at 25 °C, while the activity was lower at 10 and 5 °C. We have intentionally exposed samples collected on a sunny day to the various temperatures, as elevated temperatures are expected under these conditions. Obviously, 45 °C represents a threshold for physiological activities. A similar temperature dependence was found in an Antarctic *P. crispa*, where the Y(II) declined below 15 and above 25 °C (Kosugi et al. 2010), pointing to similar physiological traits.

## Conclusion

The present study gives a detailed overview on the physiological capabilities and ultrastructure of *P. calophylla*, a terrestrial alga that tolerates a high degree of water loss in its vegetative state. Field-collected samples showed very little sensitivity to desiccation or osmotic water reduction, enabling them to survive under extreme water-limiting conditions. The unaffected ultrastructure of the cells contributes to this high tolerance. The underlying molecular mechanisms, however, are poorly studied and, hence, *P. calophylla* would represent a perfect model system for metabolomic or transcriptomic changes under desiccation stress.

## Abbreviations

MAA: Mycosporine-like amino acid
UVR: Ultraviolet radiation
